# Acute High-Intensity Interval Exercise-Induced Redox Signaling Is Associated with Enhanced Insulin Sensitivity in Obese Middle-Aged Men

**DOI:** 10.3389/fphys.2016.00411

**Published:** 2016-09-16

**Authors:** Lewan Parker, Nigel K. Stepto, Christopher S. Shaw, Fabio R. Serpiello, Mitchell Anderson, David L. Hare, Itamar Levinger

**Affiliations:** ^1^Clinical Exercise Science Research Program, Institute of Sport, Exercise and Active Living, Victoria UniversityMelbourne, VIC, Australia; ^2^Centre for Physical Activity and Nutrition Research, School of Exercise and Nutrition Sciences, Deakin UniversityGeelong, VIC, Australia; ^3^University of Melbourne and the Department of Cardiology, Austin HealthMelbourne, VIC, Australia

**Keywords:** redox signaling, insulin sensitivity, obesity, oxidative stress, high-intensity exercise, stress kinase

## Abstract

**Background:** Obesity and aging are associated with increased oxidative stress, activation of stress and mitogen activated protein kinases (SAPK), and the development of insulin resistance and metabolic disease. In contrast, acute exercise also increases oxidative stress and SAPK signaling, yet is reported to enhance insulin sensitivity and reduce the risk of metabolic disease. This study explored this paradox by investigating the effect of a single session of high-intensity interval-exercise (HIIE) on redox status, muscle SAPK and insulin protein signaling in eleven middle-aged obese men.

**Methods:** Participants completed a 2 h hyperinsulinaemic-euglycaemic clamp at rest, and 60 min after HIIE (4 × 4 mins at 95% HR_peak_; 2 min recovery periods), separated by 1–3 weeks.

**Results:** Irrespective of exercise-induced changes to redox status, insulin stimulation both at rest and after HIIE similarly increased plasma superoxide dismutase activity, plasma catalase activity, and skeletal muscle 4-HNE; and significantly decreased plasma TBARS and hydrogen peroxide. The SAPK signaling pathways of p38 MAPK, NF-κB p65, and JNK, and the distal insulin signaling protein AS160^Ser588^, were activated with insulin stimulation at rest and to a greater extent with insulin stimulation after a prior bout of HIIE. Higher insulin sensitivity after HIIE was associated with higher insulin-stimulated SOD activity, JNK, p38 MAPK and NF-κB phosphorylation (*r* = 0.63, *r* = 0.71, *r* = 0.72, *r* = 0.71; *p* < 0.05, respectively).

**Conclusion:**These findings support a role for redox homeostasis and SAPK signaling in insulin-stimulated glucose uptake which may contribute to the enhancement of insulin sensitivity in obese men 3 h after HIIE.

## Introduction

Obesity is a major risk factor for insulin resistance and type 2 diabetes. Regular physical activity improves glycemic control and as such is a key lifestyle goal for the prevention and management of obesity and type 2 diabetes (Malin and Braun, 2016). Even a single session of exercise can enhance insulin sensitivity in the hours after exercise (Frosig and Richter, 2009). However, the mechanisms by which exercise improves insulin sensitivity are not completely understood, but may include oxidation-reduction (redox) reactions and their inherent capacity to both impair and/or facilitate insulin signaling and insulin-stimulated glucose uptake (Tiganis, 2011).

Oxidative stress is defined as an imbalance between reactive oxygen species (ROS) production and endogenous antioxidant defenses (Radak et al., 2013). While modest increases in ROS are regulated by endogenous antioxidants, certain ROS-inducing stimuli, such as exercise, diet, age, and disease, may overpower these systems in favor of oxidative stress (Valko et al., 2007). Chronic systemic oxidative stress is associated with obesity, insulin resistance, and type 2 diabetes (Valko et al., 2007; Bashan et al., 2009). Oxidative stress-induced insulin resistance can occur through protein modification via both lipid peroxidation and the activation of stress and mitogen activated protein kinase signaling (SAPK) pathways (Tanti and Jager, 2009; Tiganis, 2011). Sustained activation of these redox-sensitive signaling pathways leads to inhibitory phosphorylation of the insulin receptor substrate 1 (IRS-1) at human serine residues 312 and 307, promoting IRS-1 degradation, impaired insulin signaling, and attenuation of insulin-stimulated glucose uptake (de Alvaro et al., 2004; Werner et al., 2004; Gual et al., 2005; Archuleta et al., 2009; Tanti and Jager, 2009; Pillon et al., 2012). The prevention of IRS-1 degradation through the inhibition of ROS and/or SAPK signaling has been shown to restore insulin signaling and insulin-stimulated glucose uptake (Somwar et al., 2000; Geiger et al., 2005; Tiganis, 2011; Pillon et al., 2012). Collectively, these studies suggest a pathological role of redox induced lipid peroxidation and SAPK signaling in aberrant insulin signaling and subsequent insulin resistance.

Paradoxically, ROS produced during and after muscular contraction also transiently activate SAPK signaling pathways and lipid peroxidation (Kramer and Goodyear, 2007), however during this period glucose uptake and insulin sensitivity are reported to be enhanced (Frosig and Richter, 2009). An accumulation of research now suggests that depending on the biological context, redox signaling is integral for optimal physiological functioning and adaptation to physiological stress (Radak et al., 2013). Redox-signaling may play an important role in contraction-induced (Sandström et al., 2006) and insulin-stimulated glucose uptake (Kim et al., 2006; Bashan et al., 2009; Loh et al., 2009; Trewin et al., 2015); whether these redox-signaling pathways are activated with insulin stimulation after a single session of exercise are unknown. High-intensity interval exercise (HIIE) is an effective exercise mode for improving glycemic control in clinical populations (Gibala et al., 2012; Liubaoerjijin et al., 2016), however the impact of acute HIIE on redox-sensitive protein signaling and insulin sensitivity in obese middle-aged males is unknown. The aim of this study was to test the hypothesis that a single session of HIIE would transiently increase oxidative stress and SAPK signaling and insulin signaling which may, at least in part, be related to post-exercise enhancement of insulin sensitivity in middle-aged, obese males.

## Materials and methods

### Participants

Eleven middle-aged (58.1 ± 2.2 years mean ± SEM, range 40–70 years), obese men (BMI = 33.1 ± 1.4 kg·m^−2^), without diabetes (fasting glucose: 5.3 ± 0.2 mmol·L^−1^ and HbA1_C_: 5.6 ± 0.1%; 34 ± 1.1 mmol/mol), participated in the study (Levinger et al., 2014). Verbal and written explanations about the study were provided to participants prior to providing written consent. Exclusion criteria for participation included medications known to affect insulin secretion and/or insulin sensitivity; musculoskeletal or other conditions that prevent daily activity; and symptomatic or uncontrolled metabolic or cardiovascular disease. This study was approved by the Victoria University Human Research Ethics Committee and carried out in accordance with The Code of Ethics of the World Medical Association (Declaration of Helsinki) for experiments involving humans.

### Screening and preliminary testing

Participants were asked to complete a symptom limited incremental cycle $\dot{V}$O_2peak_ determination test as previously described (Levinger et al., 2007). Ventilatory expired gas (15 second averages) was collected from each participant and analyzed using a metabolic cart (Medgraphics, Cardio2 and CPX/D System, USA).

### Study design

Participants abstained from food (overnight fast), physical activity (72 h), and alcohol and caffeine consumption (24 h) prior to each trial day. To avoid glycogen depletion volunteers were provided dietary information and asked to consume approximately 300 g of carbohydrate 24 h prior to their first trial. This was recorded in a diet diary and replicated for their second trial. For the main experimental trial participants completed a 2 h hyperinsulinaemic-euglycaemic clamp (insulin clamp) at rest (rest trial), and 60 min after HIIE (exercise trial), separated by 1–3 weeks.

### Rest trial

Participants arrived in the morning following an overnight fast and a 2 h insulin clamp was performed to measure baseline insulin sensitivity via methods previously reported (Hutchison et al., 2011; Stepto et al., 2013; Levinger et al., 2014). In brief, insulin (Actrapid; Novo Nordisk, Bagsvaerd, Denmark) was infused at 40 mU.m^−2^ per minute for 120 min inducing a stable state of hyperinsulinemia (Levinger et al., 2014). Concomitantly, exogenous glucose was infused at a rate necessary to maintain a stable blood glucose reading of ~5 mmol.l^−1^ which was assessed every 5 min during the insulin clamp with an automated analyzer (YSI 2300 STAT Plus Glucose & Lactate Analyzer, USA). Insulin sensitivity was calculated by averaging the glucose infusion rate (GIR, mg·kg^−1^·min^−1^) over the final 30 min of the insulin clamp and normalized to serum insulin levels (*M*-Value) (Howlett et al., 2008). Muscle biopsies and venous blood samples were taken at baseline and after the 2 h insulin clamp (~3 h after exercise).

### Exercise trial

Approximately 1–3 weeks later participants arrived after an overnight fast and performed 30 min of HIIE on a Lode cycle ergometer (Corvial, Lode B.V., Groningen, The Netherlands). The HIIE included a 4 min warm-up at 50–60% HR_peak_, followed by 4 × 4 min cycling bouts at 95% HR_peak_, interspersed with 2 min active recovery at 50–60% HR_peak_. The target HR for the exercise session was determined by the heart rate reserve method using the following formula: Exercise target HR = % of target intensity (HR_peak_–HR_rest_) + HR_rest_. After the exercise bout participants underwent 1 h of passive recovery after which the 2 h insulin clamp was performed as per the previous trial.

### Skeletal muscle and blood sampling

Vastus lateralis muscle and venous blood samples were taken at baseline and after the insulin clamp in the rest trial; and at baseline, pre-insulin clamp (1 h post-exercise) and post-insulin clamp (~3 h post-exercise) in the exercise trial. Muscle samples were obtained from the vastus lateralis under local anesthesia (xylocaine 1%) utilizing a Bergström needle with suction (Evans et al., 1982). The samples were immediately frozen in liquid nitrogen and stored at −80°C until analysis. Venous blood was collected from an antecubital vein (contra-lateral to infusions) via an intravenous cannula and appropriate collection tubes. Blood samples were centrifuged at 3500 rpm for 15 min at 4°C and plasma subsequently aliquoted and stored at −80°C until analyzed.

### Plasma redox status analysis

Plasma thiobarbituric acid reactive substances (TBARS; Cayman Chemical, Ann Arbor, MI, USA), Catalase activity (Cayman Chemical, Ann Arbor, MI, USA), Superoxide Dismutase (SOD) activity (Cayman Chemical, Ann Arbor, MI, USA), and Hydrogen Peroxide (Amplex UltraRed assay; Molecular Probes, Eugene, Oregon, USA) were analyzed by a spectrophotometer (xMark microplate spectrophotometer, Bio-Rad Laboratories, Mississauga, ON, Canada) in duplicate as per the manufacturer's instructions. One unit of catalase activity is defined as the amount of enzyme required to cause the formation of 1.0 nmol of formaldehyde per minute at 25°C. One unit of SOD activity is defined as the amount of enzyme needed to exhibit 50% dismutation of the superoxide radical. TBARS is expressed in nmol/ml of malondialdehyde equivalents. Plasma hydrogen peroxide is expressed in nmol/ml. Intra-assay coefficients of variation were determined from each duplicate for all participants and resulted in a coefficient of variation of 1.05, 1.99, 4.91, and 1.88% for TBARS, SOD, catalase and hydrogen peroxide, respectively. Inter-assay coefficients of variation for assay standards were 0.65, 3.65, 2.30, 1.20, for TBARS, SOD, catalase, and hydrogen peroxide, respectively.

### Skeletal muscle protein analysis

Abundance of specific proteins in muscle samples were determined with all constituents present (i.e., no centrifugation) (Murphy and Lamb, 2013). Thirty cryosections of skeletal muscle (20 μm) were cut (Cryostat HM550, Thermo Scientific, Australia) and homogenized with 300 μl of homogenizing buffer (0.125 M TRIS-HCL (pH 6.8), 4% SDS, 10% Glycerol, 10 mM EGTA, and 0.1 M DTT). Samples were rotated for 1 h at room temperature, vortexed and frozen at −80°C. Samples were then thawed on ice and protein content was determined using the Red 660 Protein Assay (G-Biosciences, St. Louis, MO, USA) as per the manufacturer's instructions. Eight Microgram of protein was prepared in 3 μl of Bromophenol blue (1%), heated for 5 min at 95°C and separated by 7.5% Criterion™ TGX™ Pre-Cast Gels. The separated proteins were transferred to a membrane (Trans-Blot Turbo PVDF Transfer Pack, Bio-Rad, Richmond, CA) with a Trans-Blot Turbo Transfer System (Bio-Rad). Membranes were blocked with Tris-Buffered Saline-Tween (TBST: 0.1 M Tris Base, 1.5 M NaCl, 0.1% Tween-20) and 5% skim milk for 1 h and then washed (4 × 5 min) with TBST. Membranes were incubated at 4°C overnight with the following primary antibodies: Phospho-SAPK/JNK^Thr183/Tyr185^ (Cell Signaling Technology; CST #9251), SAPK/JNK (CST #9252), phospho-p38 MAPK^Thr180/Tyr182^ (CST #9211), p38 MAPK (CST #9212), phospho-NF-κB p65^Ser536^ (CST #3033), NF-κB p65 (CST #8242), IκBα (CST #4814), phospho-PKC δ/θ^Ser643/676^ (CST #9376), phospho-IRS-1^Ser307^ (CST #2384), phospho-AS160^Ser318^ (CST #8619), phospho-AS160^Ser588^ (CST #8730), phospho-GSK-3α/β^Ser21/9^ (CST #9331), AS160 (CST #2447), 4-Hydroxynonenal (Abcam, ab46545), and IRS-1 (Millipore, 06-248). After incubation, membranes were washed with TBST, incubated at room temperature (1 h) with horseradish peroxidase conjugated secondary antibody, re-washed and incubated in SuperSignal West Femto Maximum Sensitivity substrate for 5 min, and protein densitometry measured via ChemiDoc™ MP System (Bio-Rad) and Image Lab software (Bio-Rad). Membranes were then washed briefly in TBST, stained with 0.1% Brilliant Blue R-350 (Sigma Aldrich, USA) in 1:1 methanol/distilled water (dH_2_O) solution for 3 min, de-stained in 1:5:4 ethanol/acetic acid/dH_2_O solution for 40 s, and rinsed with dH_2_O and air-dried for ~1 h prior to imaging. Densitometry values are expressed relative to a pooled internal standard and normalized to the total protein content of each lane obtained from the modified Coomassie staining protocol. Where appropriate, phosphorylated proteins are expressed relative to total specific protein content.

### Statistical analysis

Data were checked for normality and analyzed using Predictive Analytics Software (PASW, USA). Comparisons of means at baseline and after insulin stimulation in the rest and exercise trial were examined using a two way (intervention x time point) repeated measures analysis of variance (ANOVA). Comparisons of multiple means within the exercise trial were examined using a one way repeated measures ANOVA. *Post-hoc* analysis were conducted using Fisher's LSD. Associations between insulin sensitivity, redox status and SAPK signaling were analyzed using Pearson's coefficient of correlation. Cook's Distance was used as a measure of influence where observations greater than one Cook's D were excluded from correlation analysis (Cook, 1979). All data are reported as mean ± standard error of mean (SEM) and all statistical analysis were conducted at the 95% level of significance (*p* < 0.05). Trends were reported when *p*-values were greater than 0.05 and less than 0.1.

## Results

### Insulin sensitivity

Previously reported, exercise significantly increased insulin sensitivity (glucose infusion rate and *m*-value) by ~34–40% compared to the rest trial (Levinger et al., 2014).

### Plasma redox status

**Figure 1:**
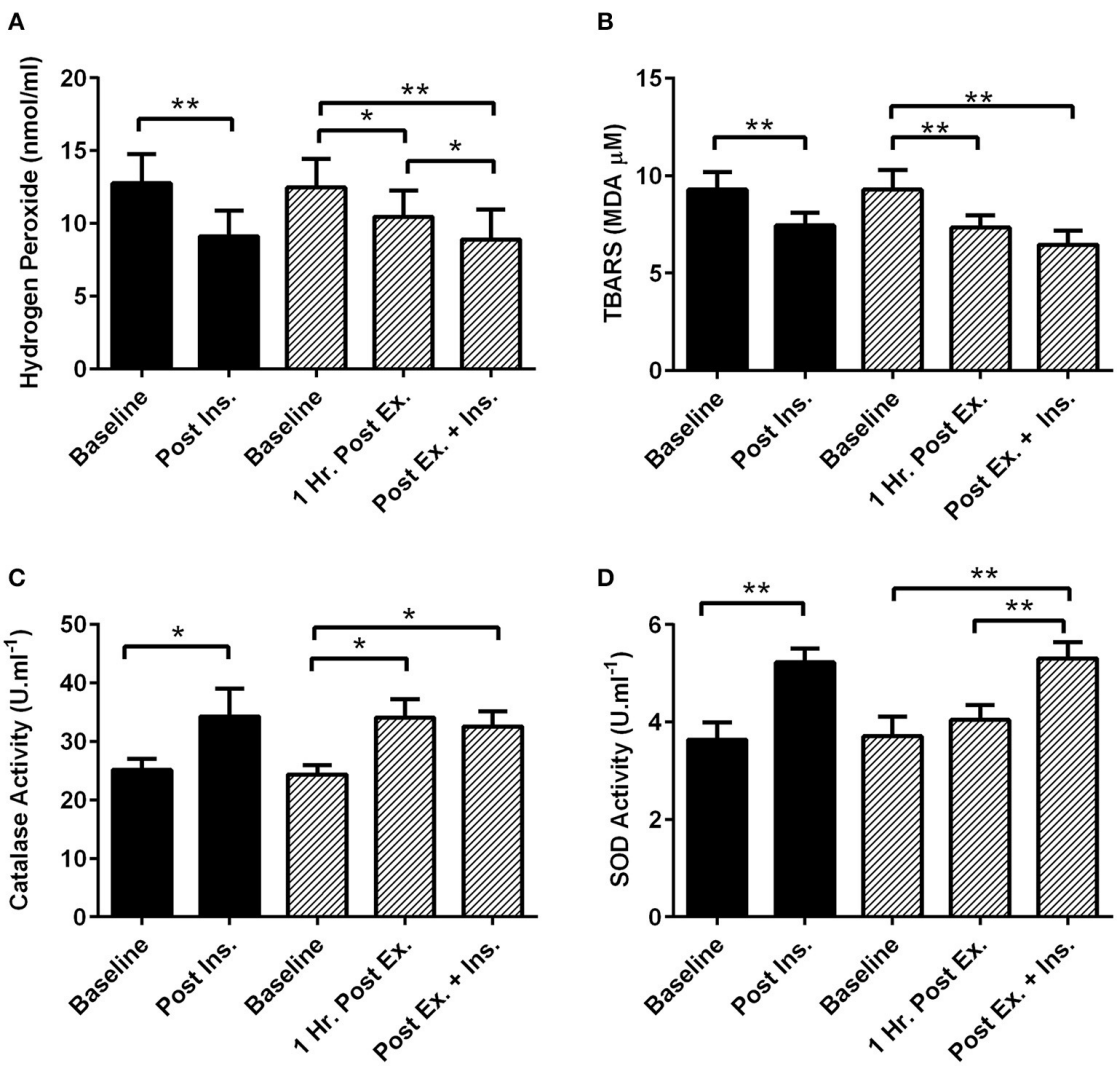
**Exercise, insulin stimulation, and plasma redox status**. Oxidative stress and antioxidant enzymatic activity biomarkers in plasma 1 h after exercise (prior to insulin stimulation), and pre and post insulin stimulation at rest and 3 h after exercise (*n* = 11). **(A)** Hydrogen Peroxide; **(B)** Thiobarbituric acid reactive substances (TBARS); **(C)** Catalase activity; **(D)** Superoxide dismutase activity (SOD). *p < 0.05 and **p < 0.01 are significantly different. Black bars = rest trial. Diagonal line bars = exercise trial. Ex. = High-intensity interval exercise. Ins. = insulin stimulation via the hyperinsulinaemic-euglycaemic clamp.

One hour after the HIIE session (prior to insulin stimulation) catalase activity significantly increased and TBARS and hydrogen peroxide decreased (Figure 1). Insulin stimulation both at rest and after exercise elicited a similar decrease in plasma hydrogen peroxide and TBARS, and increase in plasma catalase and superoxide dismutase activity (Figure 1).

### Skeletal muscle SAPK signaling

**Figure 2:**
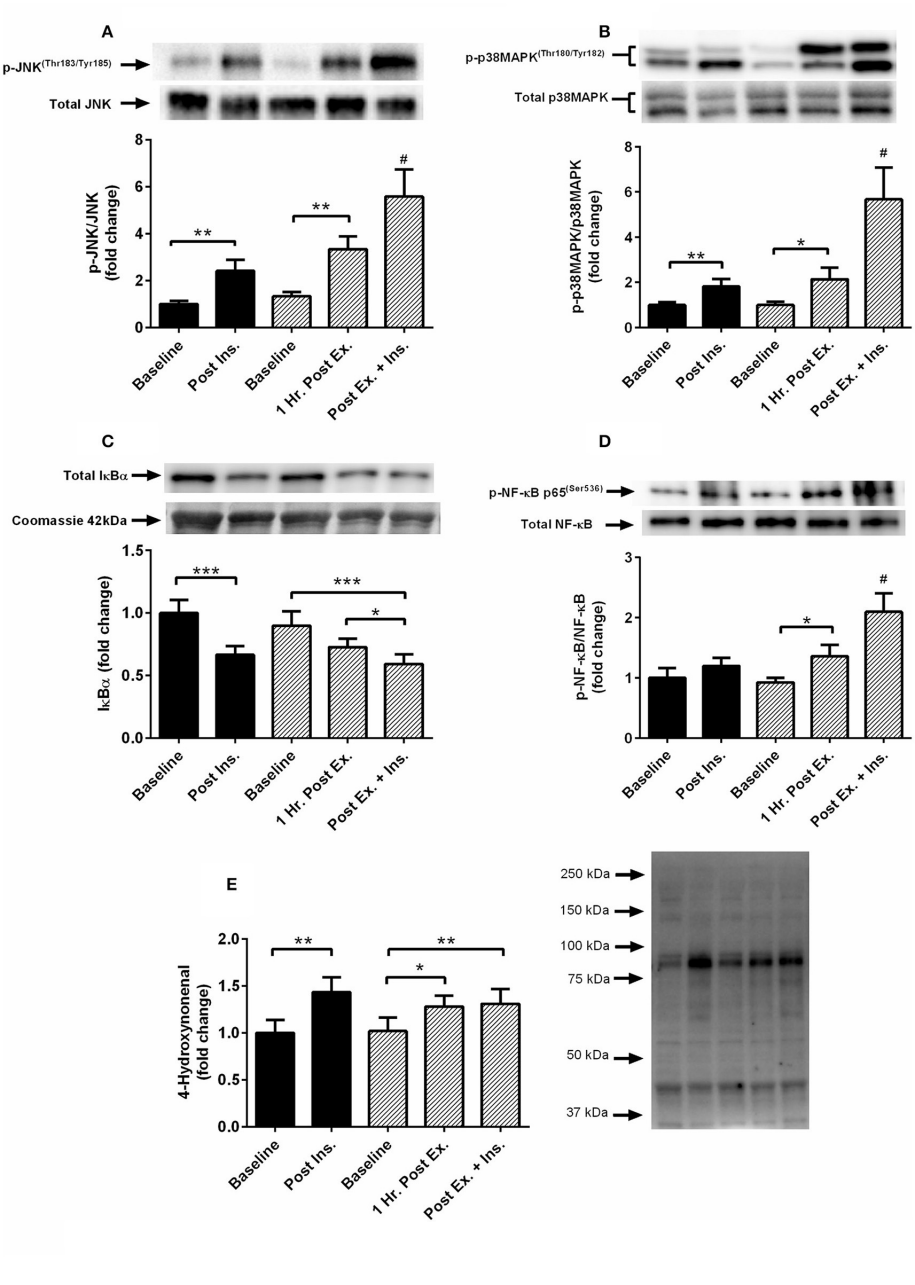
**Exercise, insulin stimulation, and skeletal muscle SAPK signaling**. Phosphorylation relative to total protein content in skeletal muscle 1 h after exercise (prior to insulin stimulation), and pre and post insulin stimulation at rest and 3 h after exercise (*n* = 11). **(A)** JNK^(Thr183/Tyr185^); **(B)** p38 MAPK^(Thr180/Tyr182)^; **(C)** IκBα; **(D)** NF-κB p65^(Ser536)^; **(E)**: 4-hydroxynonenal. *p < 0.05, **p < 0.01, ***p < 0.001 and # is significantly different to all time-points in both the rest and exercise trial. Black bars = rest trial. Diagonal line bars = exercise trial. Ex. = High-intensity interval exercise. Ins. = insulin stimulation via the hyperinsulinaemic-euglycaemic clamp.

**Figure 3:**
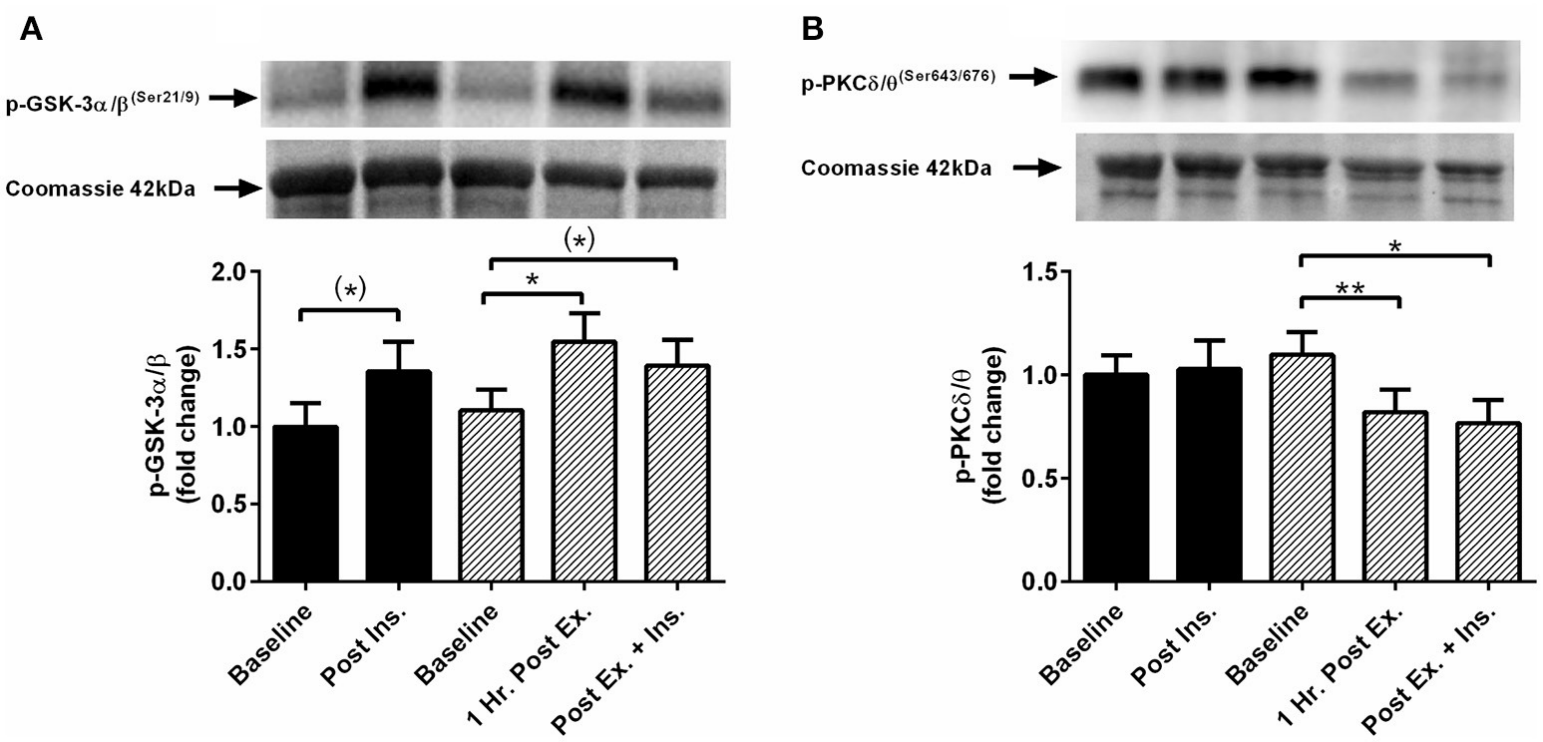
**Exercise, insulin stimulation, and skeletal muscle glycogen synthase kinase and protein kinase C signaling**. Phosphorylation relative to total protein content in skeletal muscle 1 h after exercise (prior to insulin stimulation), and pre and post insulin stimulation at rest and 3 h after exercise (*n* = 11). **(A)** GSK-3α/β^(Ser21/9)^; **(B)** PKC δ/θ^(Ser643/676)^. *p < 0.05 and **p < 0.01 are significantly different. Black bars = rest trial. Diagonal line bars = exercise trial. Ex. = High-intensity interval exercise. Ins. = insulin stimulation via the hyperinsulinaemic-euglycaemic clamp.

The acute session of exercise (prior to insulin stimulation) significantly increased phosphorylation of JNK^Thr183/Tyr185^, p38 MAPK^Thr180/Tyr182^, NF-κB p65^Ser536^, GSK-3α/β^Ser21/9^, and 4-HNE protein modification (Figures 2, 3). In contrast, phosphorylated PKC δ/θ^Ser643/676^ was significantly lower after exercise (Figure 3). Insulin stimulation in the rest trial significantly increased phosphorylation of JNK^Thr183/Tyr185^, p38 MAPK^Thr180/Tyr182^, and 4-HNE protein modification (Figure 2). The prior bout of HIIE significantly increased insulin-stimulated phosphorylation of JNK, p38 MAPK, and NF-κB p65 to a greater extent. PKC δ/θ phosphorylation remained lower compared to baseline. Total protein content of IκBα was significantly lower after insulin stimulation in both the rest and HIIE trial (Figure 2). There was a tendency for increased phosphorylation of GSK-3α/β after insulin stimulation in both the rest and exercise trial.

### Skeletal muscle insulin signaling

**Figure 4:**
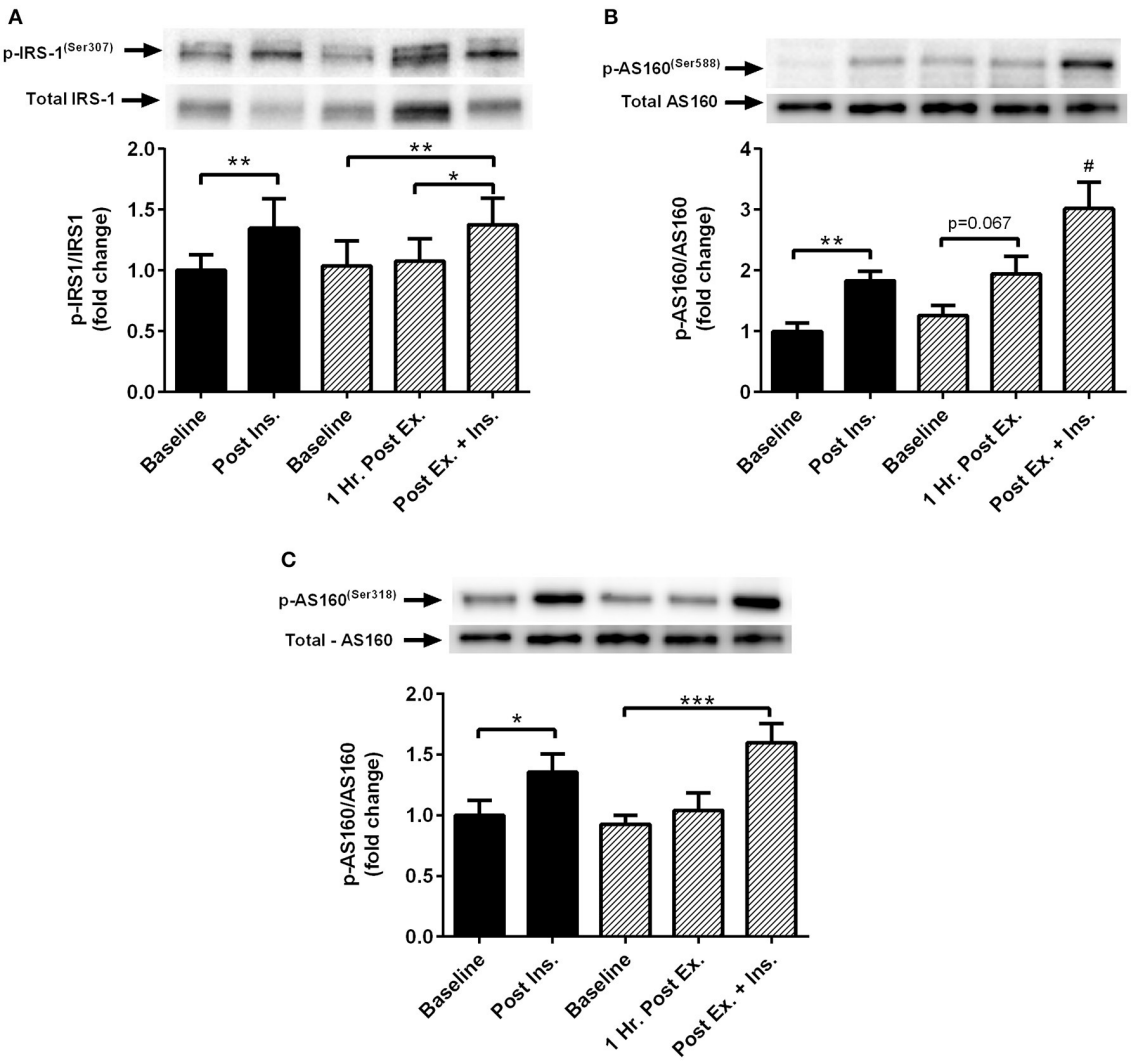
**Exercise, insulin stimulation and skeletal muscle insulin signaling**. Phosphorylation relative to total protein content of insulin signaling proteins in skeletal muscle 1 h after exercise (prior to insulin stimulation), and pre and post insulin stimulation at rest and 3 h after exercise (*n* = 11). **(A)** IRS-1^(ser307)^; **(B)** AS160^(ser588)^; **(C)** AS160^(ser318)^. *p < 0.05, **p < 0.01, ***p < 0.001 and # is significantly different to all time-points in both the rest and exercise trial. Black bars = rest trial. Diagonal line bars = exercise trial. Ex. = High-intensity interval exercise. Ins. = insulin stimulation via the hyperinsulinaemic-euglycaemic clamp.

Insulin stimulation both at rest and after HIIE elicited a similar increase in phosphorylation of IRS-1^Ser307^ and AS160^Ser318^ (Figure 4). There was a tendency for increased AS160^Ser588^ phosphorylation after HIIE (Figure 4). Insulin stimulated AS160^Ser588^ phosphorylation was greater after a prior bout of HIIE.

### Insulin sensitivity correlations

Insulin sensitivity (*m*-value) following exercise was positively associated with higher levels of insulin-stimulated SOD activity (*r* = 0.634, *p* = 0.036, *n* = 11), phosphorylated-JNK (*r* = 0.709, *p* = 0.007, *n* = 10), p38 MAPK (*r* = 0.724, *p* = 0.018, *n* = 10) and NF-κB p65 (*r* = 0.708, *p* = 0.022, *n* = 10), and tended to correlate with lower levels of phosphorylated PKC δ/θ (*r* = −0.571, *p* = 0.066, *n* = 11). No correlations between insulin sensitivity and the variables of interest were detected in the rest trial (*p* > 0.05).

## Discussion

We report the novel finding that insulin-stimulated phosphorylation of p38 MAPK, NF-κB p65, and JNK occurred to a greater extent after a prior bout of HIIE in obese middle-aged males. Furthermore, increased SAPK signaling coincided with enhanced insulin signaling and whole body insulin sensitivity, indicating a potential role for SAPK signaling in the post-exercise enhancement of insulin sensitivity. We also reported that insulin stimulation increased plasma antioxidant activity, decreased plasma oxidative stress, and increased 4-HNE in skeletal muscle to a similar extent, irrespective of exercise-induced changes in redox status.

## Prior exercise, insulin stimulation, and SAPK and insulin signaling

We report for the first time that in middle-aged obese males, p38 MAPK, NF-κB p65, and JNK signaling pathways are activated with insulin stimulation at rest, and to a greater extent with insulin stimulation after a prior bout of HIIE. Surprisingly, increased p38 MAPK, NF-κB p65, and JNK signaling did not coincide with greater IRS-1^(ser307)^ phosphorylation, decreased phosphorylation of AS160^(ser318, ser588)^, or impaired insulin sensitivity. Sustained activation of 4-HNE, NF-κB, JNK, p38 MAPK, and PKC signaling have been linked to attenuated insulin action and signaling via IRS-1 serine phosphorylation (de Alvaro et al., 2004; Gual et al., 2005; Archuleta et al., 2009; Pillon et al., 2012). In contrast, the transient activation of SAPK signaling pathways may augment insulin signaling and insulin-stimulated glucose uptake (Somwar et al., 2000; Thong et al., 2003; Geiger et al., 2005; Kim et al., 2006; Sandström et al., 2006; Berdichevsky et al., 2010). Our findings support redox sensitive SAPK signaling as a regulator of *in vivo* insulin-stimulated glucose uptake which may, at least in part, contribute to the post-exercise enhancement of insulin sensitivity.

We report similar insulin stimulated phosphorylation of IRS-1^(ser307)^ both at rest and after a prior bout of HIIE. Insulin-stimulated phosphorylation of IRS-1^ser307^ may occur due to feedback inhibition of the insulin signaling cascade reported during conditions of hyperinsulinemia (Lee et al., 2003; Werner et al., 2004). Alternatively, IRS-1^(ser307)^ phosphorylation may be necessary for insulin-stimulated propagation of the insulin signaling cascade and glucose uptake (Danielsson et al., 2005). Regardless of the biological role, IRS-1^(ser307)^ phosphorylation was similar between rest and exercise trials and therefore unlikely to be contributing to the post-exercise enhancement of insulin sensitivity. These findings support previous reports that post-exercise enhancement of insulin sensitivity likely occurs downstream in the insulin signaling cascade (Frosig and Richter, 2009; Castorena et al., 2014).

p38 MAPK can be activated by both insulin and contraction of skeletal muscle, and may play a role in glucose metabolism (Somwar et al., 2000; Thong et al., 2003). Current cell culture and animal research is divided with research reporting a role for p38 MAPK in both insulin sensitivity (Somwar et al., 2000; Geiger et al., 2005) and insulin resistance (Diamond-Stanic et al., 2011). The discrepancy in findings may be the result of non-specific p38 MAPK inhibitors which can influence metabolism through modification of AKT, GSK-3 and AMPK signaling pathways independent of p38 MAPK (Kim et al., 2006; Diamond-Stanic et al., 2011). However, even when a specific p38 MAPK inhibitor was used phosphorylation/activity of p38 MAPK was either not measured or did not change under the conditions of interest (Diamond-Stanic et al., 2011). In humans, a recent study found a small but significant reduction (~6%) in post-exercise insulin sensitivity with the infusion of the antioxidant n-acetylcysteine (Trewin et al., 2015). Although p38 MAPK phosphorylation was decreased immediately after exercise with n-acetylcysteine infusion, phosphorylation 3 h after exercise and a further 2 h later after insulin stimulation were not different to baseline levels or the placebo intervention (Trewin et al., 2015). In contrast, Thong et al. (2003) reported p38 MAPK phosphorylation in human skeletal muscle to be increased 3 h after one-legged knee extensor exercise which was further phosphorylated after insulin stimulation. In support of the insulin sensitizing role of p38 MAPK in humans, we measured increased phosphorylation with insulin stimulation at rest, 1 h after exercise (prior to insulin stimulation), and the greatest phosphorylation with insulin stimulation after the prior bout of HIIE. The discrepancy in findings between Trewin et al. (2015) and ours may be due to differences in exercise intensity (55 min at 65%$\dot{V}$O_2peak_ vs. HIIE), the participants' age (young vs. middle-aged), level of physical activity (recreationally active vs. inactive), body composition (BMI 24.8 vs. 33.1 kg·m^−2^), and muscle biopsy sampling times.

The redox sensitive JNK pathway can be activated by acute exercise and has been suggested to play a role in insulin signaling and glucose metabolism (Ropelle et al., 2006; Berdichevsky et al., 2010; Tiganis, 2011). In contrast to the current findings, Castorena et al. (2014) reported enhanced insulin sensitivity 3 h after exercise in both high fat diet and low fat diet fed rats, despite minimal effect on JNK and IRS-1^(ser307)^ phosphorylation. Furthermore, Ropelle et al. (2006) found that a single session of exercise in male rats reversed diet-induced insulin resistance 16 h later which coincided with decreased JNK, IκB–NF-κB, and IRS-1 serine phosphorylation. In support of the findings of the current study, Berdichevsky et al. (2010) reported that both chronic oxidative stress (1 μM of hydrogen peroxide for 48 h) and acute oxidative stress (500 μM of hydrogen peroxide for 3 h) similarly increased JNK phosphorylation in insulin resistant muscle cell lines. Remarkably, chronic oxidative stress increased IRS-1^(ser307)^ phosphorylation and insulin resistance whereas acute oxidative stress rescued insulin sensitivity and insulin signaling through redistribution of active cytoplasmic JNK into the nucleus. The effect of JNK and NF-κB protein signaling on the enhancement and/or impairment of insulin sensitivity is likely spatial-temporal sensitive. The current findings support a potential insulin sensitizing role of exercise-induced JNK phosphorylation in overweight, middle-aged males, however further mechanistic research is required to confirm these findings.

In the absence of changes in proximal insulin signaling, exercise-induced SAPK signaling may influence insulin sensitivity through downstream insulin signaling events. Indeed, elevated ROS production caused by glutathione peroxidase-1 knockout mice results in increased phosphorylation of AKT^(Ser473)^ and enhanced insulin sensitivity 60 min after treadmill exercise (Loh et al., 2009). Furthermore, acute hydrogen peroxide exposure (500 μM for 3 h) in C2C12 myoblasts increases insulin-stimulated glucose uptake alongside increased phosphorylation of JNK, AKT^(Ser473)^, AKT^(Thr308)^, and decreased GSK3-α/β activity (Berdichevsky et al., 2010). In contrast, isolated skeletal muscle of lean Zucker rats incubated in hydrogen peroxide (90 μM for 2 h) is reported to increase p38 MAPK phosphorylation while concomitantly decreasing AKT^(Ser473)^ phosphorylation and insulin sensitivity (Dokken et al., 2008). In the present study, we found increased insulin-stimulated SAPK signaling after exercise to coincide with enhanced insulin sensitivity and increased phosphorylation of AS160^(Ser588)^ (Levinger et al., 2014). Modulation of glycogen synthesis by oxidative stress-induced SAPK signaling is also suggested to mediate glucose metabolism (Dokken et al., 2008; Berdichevsky et al., 2010). However, similar insulin-stimulated GSK-3α/β^(Ser21/9)^ phosphorylation at rest and post-HIIE suggests this as an unlikely pathway for post-exercise enhancement of insulin sensitivity.

Protein kinase C has multiple isoforms which are suggested to play a role in insulin-stimulated glucose uptake (Frosig and Richter, 2009; Tiganis, 2011). The novel PKC δ/θ isoforms are sensitive to both change in redox status and the concentration of the lipid intermediate diacylglycerol (Ragheb et al., 2009). PKC δ/θ activation impairs insulin signaling through serine phosphorylation of IRS-1 (Greene et al., 2004; Li et al., 2004). Interestingly, phosphorylated PKC δ/θ was attenuated 1 h after HIIE and continued to be so 3 h later after insulin stimulation. This may be clinically important as obese individuals exhibit elevated levels of plasma free fatty acids (Boden, 2008) and increased PKC activity in response to insulin (Li et al., 2004). Indeed, lower insulin-stimulated PKC δ/θ phosphorylation in the exercise trial tended to correlate with higher insulin sensitivity, however phosphorylated IRS-1^(Ser307)^ was similar between the rest and exercise trial. SAPK signaling, PKC δ/θ activity, and insulin resistance often occur concomitantly (Ragheb et al., 2009). In the present study, SAPK signaling increased, PKC δ/θ phosphorylation decreased, and insulin sensitivity was enhanced after HIIE, suggesting independent pathways for PKC δ/θ and SAPK phosphorylation post-HIIE. Thus, PKC δ/θ phosphorylation may be a good candidate for further investigation with respect to post-HIIE enhanced insulin sensitivity in humans.

## Redox status and insulin signaling

The important role of redox biology in promoting and/or attenuating insulin sensitivity is well established in non-human models (Tiganis, 2011). We provide evidence that insulin stimulation increases plasma antioxidant capacity, decreases plasma oxidative stress, and increases 4-HNE in human skeletal muscle. Furthermore, this insulin stimulated shift in redox status also occurred with insulin stimulation after acute HIIE. Interestingly, the redox shift elicited by exercise (prior to insulin stimulation) had minimal effect on insulin stimulated redox status. Mahadev et al. (2004) reported that insulin stimulation increases hydrogen peroxide via increased NADPH oxidase activity, promoting insulin signaling and glucose uptake in part via decreased protein tyrosine phosphatase activity. Furthermore, previous research has shown that hydrogen peroxide can both attenuate and/or enhance insulin-stimulated glucose uptake *in-vitro* depending on its concentration (Iwakami et al., 2011). It is possible that redox status may be regulated by endogenous antioxidant defenses to maintain a redox environment conducive for optimal insulin signaling and glucose uptake. Indeed, alteration of redox homeostasis through exogenous antioxidants in humans are reported to attenuate the benefits of both acute and regular exercise on insulin sensitivity (Ristow et al., 2009; Trewin et al., 2015). We provide novel *in vivo* evidence in humans to support the important role of ROS and redox homeostasis in insulin-stimulated glucose uptake (Bashan et al., 2009; Tiganis, 2011).

## Acute exercise and redox status

A single session of HIIE increased plasma catalase activity and decreased plasma TBARS and hydrogen peroxide in obese middle-aged males. These findings contradict previous reports of increased systemic oxidative stress in obese individuals (Vincent et al., 2004, 2005). The discrepancy in findings are unclear, but may relate to increased antioxidant activity after HIIE, whereas previous studies have reported either no change or decreased antioxidant defense after continuous aerobic exercise (Vincent et al., 2004, 2005). Certainly, we and others have previously shown that higher-intensity exercise can elicit greater plasma antioxidant defense in untrained healthy males without incurring changes in plasma oxidative stress (Schneider et al., 2005; Parker et al., 2014). Obesity and aging is associated with higher levels of systemic oxidative stress which over time causes oxidative damage to proteins, lipids, and DNA, and the development of numerous pathological conditions (Valko et al., 2007). Transient shifts in redox homeostasis with regular exercise leads to the upregulation of antioxidant defense, reduces chronic oxidative stress and inflammation, and improves overall metabolic health (Ristow et al., 2009; Malin and Braun, 2016). We provide evidence that regular HIIE may be a beneficial exercise model for improving redox status and metabolic health in clinical populations.

ROS are beneficial and a necessary requirement for optimal physiological functioning and adaptation to exercise (Radak et al., 2013). We found 4-HNE protein modification, a marker of oxidative stress, to be increased in skeletal muscle 1 h after HIIE. This contradictory redox shift between plasma (decreased oxidative stress) and skeletal muscle (increased oxidative stress) may reflect the inability of plasma to accurately reflect skeletal muscle redox status (Veskoukis et al., 2009). Increased oxidative stress in skeletal muscle likely reflects localized cellular stress associated with muscular contraction and supports the important signaling role of ROS in the adaptation to exercise (Radak et al., 2013). Indeed, phosphorylation of redox-sensitive signaling proteins JNK, p38 MAPK, and NF-κB were significantly increased one after HIIE. Activation of these pathways with exercise is known to promote improvements in redox homeostasis, regulation of energy metabolism, muscle hypertrophy, inflammation, and gene transcription leading to cell proliferation, differentiation, and apoptosis (Kramer and Goodyear, 2007; Egan and Zierath, 2013). Our findings indicate that acute HIIE can transiently shift systemic redox status and activate redox-signaling pathways in skeletal muscle involved in adaptation to exercise in obese middle-aged men.

A potential limitation of the study is the small sample size. However, the present study was adequately powered to detect changes in insulin sensitivity and p38 MAPK phosphorylation in human skeletal muscle (Thong et al., 2003; Trewin et al., 2015). Additionally, only a single marker of oxidative stress was used to determine redox status in skeletal muscle. Measurement of multiple redox markers in skeletal muscle would allow for greater interpretation of the influence of skeletal muscle redox status under the conditions of interest. This study is limited to the measurement of muscle and plasma responses to a single session of HIIE and insulin stimulation. Future research would benefit by investigating these findings with respect to subsequent bouts of exercise over a longer period of time. The inclusion of an exercise only control trial would allow for greater understanding of the combined effects of exercise and insulin stimulation on the measured outcomes. Data presented in this study are delimited to obese middle-aged males and the specific HIIE protocol used. Further research is required to confirm these findings in other populations and different exercise protocols.

## Conclusion

In summary, we provide evidence that redox status and SAPK signaling are affected by both insulin stimulation and a single session of HIIE in obese middle-aged males. A prior bout of HIIE elicited greater insulin stimulated JNK, p38 MAPK, and NF-κB signaling which coincided with enhanced distal insulin signaling and whole body insulin sensitivity. These findings support the role of SAPK signaling in glycemic control and provide potential signaling pathways for the post-exercise enhancement of insulin sensitivity. Future research is required to explore potential mechanisms.

## Clinical trial number

ACTRN12613000706774, anzctr.org.au.

## Author contributions

LP, IL, CS, NS, DH, and MA contributed to the study design and acquirement of ethical approval. LP, IL, NS, FS and MA contributed to data collection. LP analyzed the data, interpreted the data, and drafted the initial manuscript. The remaining authors critically revised the manuscript. All authors approved the final version of the manuscript. IL and LP are guarantors of the manuscript and take full responsibility for the work as a whole, including the study design, access to data, and the decision to submit and publish the manuscript.

### Conflict of interest statement

The authors declare that the research was conducted in the absence of any commercial or financial relationships that could be construed as a potential conflict of interest.

## Abbreviations

4-HNE: 4-hydroxynonenal
AS160: akt substrate of 160 kDa
GSK-3: glycogen synthase kinase 3
HEC: hyperinsulinaemic-euglycaemic clamp
HIIE: high-intensity interval exercise
IL-6: interleukin 6
IRS-1: insulin receptor substrate 1
IκBα, nuclear factor of kappa light polypeptide gene enhancer in B-cells inhibitor: alpha
JNK: c-Jun N-terminal kinases
MAPK: mitogen activated protein kinase
NF-κB: nuclear factor kappa-light-chain-enhancer of activated B cells
PKC: protein kinase C
ROS: reactive oxygen species
SAPK: stress and mitogen activated protein kinase
SOD: superoxide dismutase
TBARS: thiobarbituric acid reactive substances.

## References

[B1] Archuleta T. L. Lemieux A. M. Saengsirisuwan V. Teachey M. K. Lindborg K. A. Kim J. S.. (2009). Oxidant stress-induced loss of irs-1 and irs-2 proteins in rat skeletal muscle: role of p38 mapk. Free Radic. Biol. Med. 47, 1486–1493. 10.1016/j.freeradbiomed.2009.08.014 19703555 PMC2767452

[B2] Bashan N. Kovsan J. Kachko I. Ovadia H. Rudich A. (2009). Positive and negative regulation of insulin signaling by reactive oxygen and nitrogen species. Physiol. Rev. 89, 27–71. 10.1152/physrev.00014.2008 19126754

[B3] Berdichevsky A. Guarente L. Bose A. (2010). Acute oxidative stress can reverse insulin resistance by inactivation of cytoplasmic jnk. J. Biol. Chem. 285, 21581–21589. 10.1074/jbc.M109.093633 20430894 PMC2898407

[B4] Boden G. (2008). Obesity and free fatty acids. Endocrinol. Metab. Clin. North Am. 37, 635–646, viii–ix. 10.1016/j.ecl.2008.06.007 18775356 PMC2596919

[B5] Castorena C. M. Arias E. B. Sharma N. Cartee G. D. (2014). Postexercise improvement in insulin-stimulated glucose uptake occurs concomitant with greater as160 phosphorylation in muscle from normal and insulin-resistant rats. Diabetes 63, 2297–2308. 10.2337/db13-1686 24608437 PMC4066340

[B6] Cook R. D. (1979). Influential observations in linear regression. J. Am. Stat. Assoc. 74, 169–174. 10.1080/01621459.1979.10481634

[B7] Danielsson A. Öst A. Nystrom F. H. Strålfors P. (2005). Attenuation of insulin-stimulated insulin receptor substrate-1 serine 307 phosphorylation in insulin resistance of type 2 diabetes. J. Biol. Chem. 280, 34389–34392. 10.1074/jbc.C500230200 16129690

[B8] de Alvaro C. Teruel T. Hernandez R. Lorenzo M. (2004). Tumor necrosis factor alpha produces insulin resistance in skeletal muscle by activation of inhibitor kappab kinase in a p38 mapk-dependent manner. J. Biol. Chem. 279, 17070–17078. 10.1074/jbc.M312021200 14764603

[B9] Diamond-Stanic M. K. Marchionne E. M. Teachey M. K. Durazo D. E. Kim J. S. Henriksen E. J. (2011). Critical role of the transient activation of p38 mapk in the etiology of skeletal muscle insulin resistance induced by low-level *in vitro* oxidant stress. Biochem. Biophys. Res. Commun. 405, 439–444. 10.1016/j.bbrc.2011.01.049 21241662 PMC3042539

[B10] Dokken B. B. Saengsirisuwan V. Kim J. S. Teachey M. K. Henriksen E. J. (2008). Oxidative stress-induced insulin resistance in rat skeletal muscle: role of glycogen synthase kinase-3. Am. J. Physiol. Endocrinol. Metab. 294, E615–E621. 10.1152/ajpendo.00578.2007 18089761

[B11] Egan B. Zierath J. R. (2013). Exercise metabolism and the molecular regulation of skeletal muscle adaptation. Cell Metab. 17, 162–184. 10.1016/j.cmet.2012.12.012 23395166

[B12] Evans W. J. Phinney S. D. Young V. R. (1982). Suction applied to a muscle biopsy maximizes sample size. Med. Sci. Sports Exerc. 14, 101–102. 7070249

[B13] Frøsig C. Richter E. A. (2009). Improved insulin sensitivity after exercise: focus on insulin signaling. Obesity 17(Suppl. 3), S15–S20. 10.1038/oby.2009.383 19927140

[B14] Geiger P. C. Wright D. C. Han D. H. Holloszy J. O. (2005). Activation of p38 map kinase enhances sensitivity of muscle glucose transport to insulin. Am. J. Physiol. Endocrinol. Metab. 288, E782–E788. 10.1152/ajpendo.00477.2004 15585585

[B15] Gibala M. J. Little J. P. Macdonald M. J. Hawley J. A. (2012). Physiological adaptations to low-volume, high-intensity interval training in health and disease. J. Physiol. 590, 1077–1084. 10.1113/jphysiol.2011.224725 22289907 PMC3381816

[B16] Greene M. W. Morrice N. Garofalo R. S. Roth R. A. (2004). Modulation of human insulin receptor substrate-1 tyrosine phosphorylation by protein kinase cdelta. Biochem. J. 378, 105–116. 10.1042/bj20031493 14583092 PMC1223928

[B17] Gual P. Le Marchand-Brustel Y. Tanti J. F. (2005). Positive and negative regulation of insulin signaling through irs-1 phosphorylation. Biochimie 87, 99–109. 10.1016/j.biochi.2004.10.019 15733744

[B18] Howlett K. F. Mathews A. Garnham A. Sakamoto K. (2008). The effect of exercise and insulin on as160 phosphorylation and 14-3-3 binding capacity in human skeletal muscle. Am. J. Physiol. Endocrinol. Metab. 294, E401–E407. 10.1152/ajpendo.00542.2007 18042670

[B19] Hutchison S. K. Stepto N. K. Harrison C. L. Moran L. J. Strauss B. J. Teede H. J. (2011). Effects of exercise on insulin resistance and body composition in overweight and obese women with and without polycystic ovary syndrome. J. Clin. Endocrinol. Metab. 96, E48–E56. 10.1210/jc.2010-0828 20926534

[B20] Iwakami S. Misu H. Takeda T. Sugimori M. Matsugo S. Kaneko S.. (2011). Concentration-dependent dual effects of hydrogen peroxide on insulin signal transduction in h4iiec hepatocytes. PLoS ONE 6:e27401. 10.1371/journal.pone.0027401 22102892 PMC3216925

[B21] Kim J. S. Saengsirisuwan V. Sloniger J. A. Teachey M. K. Henriksen E. J. (2006). Oxidant stress and skeletal muscle glucose transport: roles of insulin signaling and p38 mapk. Free Radic. Biol. Med. 41, 818–824. 10.1016/j.freeradbiomed.2006.05.031 16895802

[B22] Kramer H. F. Goodyear L. J. (2007). Exercise, MAPK, and NK-κB signaling in skeletal muscle. J. Appl. Physiol. 103, 388–395. 10.1152/japplphysiol.00085.2007 17303713

[B23] Lee Y. H. Giraud J. Davis R. J. White M. F. (2003). C-jun n-terminal kinase (jnk) mediates feedback inhibition of the insulin signaling cascade. J. Biol. Chem. 278, 2896–2902. 10.1074/jbc.M208359200 12417588

[B24] Levinger I. Goodman C. Hare D. L. Jerums G. Selig S. (2007). The effect of resistance training on functional capacity and quality of life in individuals with high and low numbers of metabolic risk factors. Diabetes Care 30, 2205–2210. 10.2337/dc07-0841 17563342

[B25] Levinger I. Jerums G. Stepto N. K. Parker L. Serpiello F. R. McConell G. K.. (2014). The effect of acute exercise on undercarboxylated osteocalcin and insulin sensitivity in obese men. J. Bone Miner. Res. 29, 2571–2576. 10.1002/jbmr.2285 24861730

[B27] Li Y. Soos T. J. Li X. H. Wu J. Degennaro M. Sun X. J.. (2004). Protein kinase c theta inhibits insulin signaling by phosphorylating IRS1 at ser(1101). J. Biol. Chem. 279, 45304–45307. 10.1074/jbc.C400186200 15364919

[B26] Liubaoerjijin Y. Terada T. Fletcher K. Boulé N. G. (2016). Effect of aerobic exercise intensity on glycemic control in type 2 diabetes: a meta-analysis of head-to-head randomized trials. Acta Diabetol. 53, 769–781. 10.1007/s00592-016-0870-0 27255501

[B28] Loh K. Deng H. Fukushima A. Cai X. Boivin B. Galic S.. (2009). Reactive oxygen species enhance insulin sensitivity. Cell Metab. 10, 260–272. 10.1016/j.cmet.2009.08.009 19808019 PMC2892288

[B29] Mahadev K. Motoshima H. Wu X. Ruddy J. M. Arnold R. S. Cheng G.. (2004). The nad(p)h oxidase homolog nox4 modulates insulin-stimulated generation of h2o2 and plays an integral role in insulin signal transduction. Mol. Cell. Biol. 24, 1844–1854. 10.1128/MCB.24.5.1844-1854.2004 14966267 PMC350558

[B30] Malin S. K. Braun B. (2016). Impact of metformin on exercise-induced metabolic adaptations to lower type 2 diabetes risk. Exerc. Sport Sci. Rev. 44, 4–11. 10.1249/JES.0000000000000070 26583801

[B31] Murphy R. M. Lamb G. D. (2013). Important considerations for protein analyses using antibody based techniques: down-sizing western blotting up-sizes outcomes. J. Physiol. 591, 5823–5831. 10.1113/jphysiol.2013.263251 24127618 PMC3872754

[B32] Parker L. McGuckin T. A. Leicht A. S. (2014). Influence of exercise intensity on systemic oxidative stress and antioxidant capacity. Clin. Physiol. Funct. Imaging 34, 377–383. 10.1111/cpf.12108 24283399

[B33] Pillon N. J. Croze M. L. Vella R. E. Soulére L. Lagarde M. Soulage C. O. (2012). The lipid peroxidation by-product 4-hydroxy-2-nonenal (4-hne) induces insulin resistance in skeletal muscle through both carbonyl and oxidative stress. Endocrinology 153, 2099–2111. 10.1210/en.2011-1957 22396448

[B34] Radak Z. Zhao Z. Koltai E. Ohno H. Atalay M. (2013). Oxygen consumption and usage during physical exercise: the balance between oxidative stress and ros-dependent adaptive signaling. Antioxid. Redox Signal. 18, 1208–1246. 10.1089/ars.2011.4498 22978553 PMC3579386

[B35] Ragheb R. Shanab G. M. Medhat A. M. Seoudi D. M. Adeli K. Fantus I. G. (2009). Free fatty acid-induced muscle insulin resistance and glucose uptake dysfunction: evidence for pkc activation and oxidative stress-activated signaling pathways. Biochem. Biophys. Res. Commun. 389, 211–216. 10.1016/j.bbrc.2009.08.106 19706288 PMC2981601

[B36] Ristow M. Zarse K. Oberbach A. Klöting N. Birringer M. Kiehntopf M.. (2009). Antioxidants prevent health-promoting effects of physical exercise in humans. Proc. Natl. Acad. Sci. U.S.A. 106, 8665–8670. 10.1073/pnas.0903485106 19433800 PMC2680430

[B37] Ropelle E. R. Pauli J. R. Prada P. O. De Souza C. T. Picardi P. K. Faria M. C.. (2006). Reversal of diet-induced insulin resistance with a single bout of exercise in the rat: The role of ptp1b and irs-1 serine phosphorylation. J. Physiol. 577, 997–1007. 10.1113/jphysiol.2006.120006 17008371 PMC1890392

[B38] Sandström M. E. Zhang S. J. Bruton J. Silva J. P. Reid M. B. Westerblad H.. (2006). Role of reactive oxygen species in contraction-mediated glucose transport in mouse skeletal muscle. J. Physiol. 575, 251–262. 10.1113/jphysiol.2006.110601 16777943 PMC1819411

[B39] Schneider C. D. Barp J. Ribeiro J. L. Belló-Klein A. Oliveira A. R. (2005). Oxidative stress after three different intensities of running. Can. J. Appl. Physiol. 30, 723–734. 10.1139/h05-151 16485522

[B40] Somwar R. Perreault M. Kapur S. Taha C. Sweeney G. Ramlal T.. (2000). Activation of p38 mitogen-activated protein kinase alpha and beta by insulin and contraction in rat skeletal muscle: potential role in the stimulation of glucose transport. Diabetes 49, 1794–1800. 10.2337/diabetes.49.11.1794 11078445

[B41] Stepto N. K. Cassar S. Joham A. E. Hutchison S. K. Harrison C. L. Goldstein R. F.. (2013). Women with polycystic ovary syndrome have intrinsic insulin resistance on euglycaemic-hyperinsulaemic clamp. Hum. Reprod. 28, 777–784. 10.1093/humrep/des463 23315061

[B42] Tanti J. F. Jager J. (2009). Cellular mechanisms of insulin resistance: role of stress-regulated serine kinases and insulin receptor substrates (irs) serine phosphorylation. Curr. Opin. Pharmacol. 9, 753–762. 10.1016/j.coph.2009.07.004 19683471

[B43] Thong F. S. Derave W. Urso B. Kiens B. Richter E. A. (2003). Prior exercise increases basal and insulin-induced p38 mitogen-activated protein kinase phosphorylation in human skeletal muscle. J. Appl. Physiol. (1985) 94, 2337–2341. 10.1152/japplphysiol.00036.2003 12611773

[B44] Tiganis T. (2011). Reactive oxygen species and insulin resistance: the good, the bad and the ugly. Trends Pharmacol. Sci. 32, 82–89. 10.1016/j.tips.2010.11.006 21159388

[B45] Trewin A. J. Lundell L. S. Perry B. D. Patil K. V. Chibalin A. V. Levinger I.. (2015). Effect of n-acetylcysteine infusion on exercise-induced modulation of insulin sensitivity and signaling pathways in human skeletal muscle. Am. J. Physiol. Endocrinol. Metab. 309, E388–E397. 10.1152/ajpendo.00605.2014 26105008

[B46] Valko M. Leibfritz D. Moncol J. Cronin M. T. D. Mazur M. Telser J. (2007). Free radicals and antioxidants in normal physiological functions and human disease. Int. J. Biochem. Cell Biol. 39, 44–84. 10.1016/j.biocel.2006.07.001 16978905

[B47] Veskoukis A. S. Nikolaidis M. G. Kyparos A. Kouretas D. (2009). Blood reflects tissue oxidative stress depending on biomarker and tissue studied. Free Radic. Biol. Med. 47, 1371–1374. 10.1016/j.freeradbiomed.2009.07.014 19616614

[B48] Vincent H. K. Morgan J. W. Vincent K. R. (2004). Obesity exacerbates oxidative stress levels after acute exercise. Med. Sci. Sports Exerc. 36, 772–779. 10.1249/01.MSS.0000126576.53038.E9 15126709

[B49] Vincent H. K. Vincent K. R. Bourguignon C. Braith R. W. (2005). Obesity and postexercise oxidative stress in older women. Med. Sci. Sports Exerc. 37, 213–219. 10.1249/01.MSS.0000152705.77073.B3 15692315

[B50] Werner E. D. Lee J. S. Hansen L. Yuan M. S. Shoelson S. E. (2004). Insulin resistance due to phosphorylation of insulin receptor substrate-1 at serine 302. J. Biol. Chem. 279, 35298–35305. 10.1074/jbc.M405203200 15199052

